# Supplementary material for: Combining Clinician Expertise with Prompt Engineering enhances Small Language Models Reliability for Cancer Entity Recognition in Electronic Health Records
Source: medRxiv. 2025 Oct 21:2025.10.16.25337917. Preprint. [Version 1] doi: 10.1101/2025.10.16.25337917 (PMC12633604; doi:10.1101/2025.10.16.25337917)
Supplement: Supplement 1 [file media-1.pdf]

Supplementary material

S1 Study cohorts

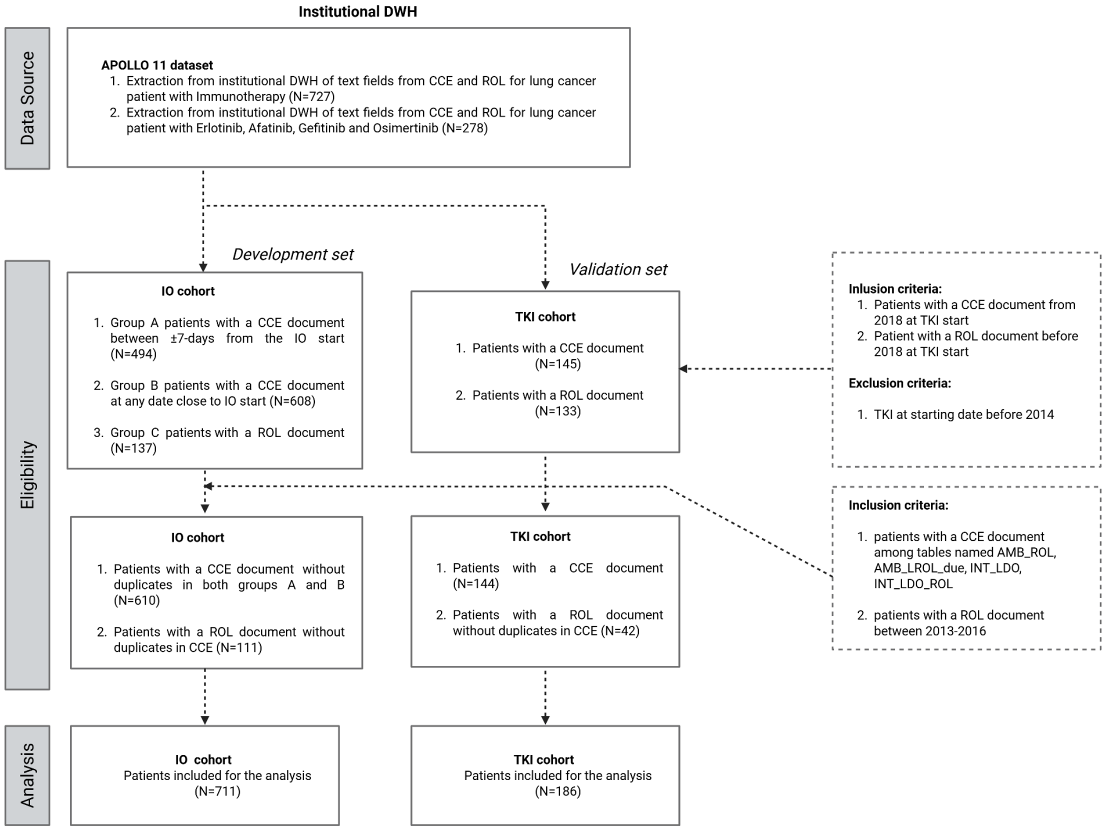

Figure S1.1: Flowchart of the patient selection from the institutional DWH. Data are split into development (IO cohort) and validation set (TKI cohort).

Grammar: Example of JSON Output

```
{ "SMOKING": "former smoker" | "current smoker" | "never smoked" | "not mentioned",
"PDL1": "<1%" | "1-49%" | ">=50%" | "not mentioned",
"HISTOLOGY": "adenocarcinoma" | "squamous" | "other histology" | "not mentioned",
"ECOG": "0" | "1" | "2" | "3" | "not mentioned",
"T_IO_START": "T0" | "T1" | "T2" | "T3" | "T4" | "not mentioned",
"N_IO_START": "N0" | "N1" | "N2" | "N3" | "not mentioned",
"M_IO_START": "M0" | "M1",
"STAGE": "III" | "IV" | "not mentioned",
"BONE_METASTASIS": boolean,
"BRAIN_METASTASIS": boolean,
"LIVER_METASTASIS": boolean }
```

Figure S2.1: JSON grammar schema for all the features selected.

Entity	Type	Labels
**Smoking history**	Multiclass	Current smoker, Former smoker, Never smoked, not mentioned
**Histological subtype**	Multiclass	Adenocarcinoma, Squamous cell carcinoma, Other histologies, Not mentioned
**ECOG PS**	Multiclass	0 (Fully active), 1 (Restricted but ambulatory), 2 (Ambulatory, self-care possible), 3 (Limited selfcare, bed/chair-bound >50%), 4 (Completely disabled), 5 (Dead).
**PD-L1 expression**	Multiclass	<1%, 1–49%, >50%, Not mentioned
**Presence of Metastases**	Binary	Bone Metastases (Ture/False), Brain Metastases (True/False), Liver Metastases(True/False)
**Tumour staging (AJCC 8th Edition)**	Multiclass	T category (T0–T4), N category (N0–N3), M category (M0–M1), Not mentioned
**Overall clinical stage**	Multiclass	Stage III, Stage IV, not mentioned

Table S2.2: Description of the cancer-related entities selected for the extraction.

S3 Benchmark of models

Table S3.1: LlaMa 3.1 8B performance for each feature extracted and prompting strategy (Italian) on the IO cohort.

Type of prompt	Metrics	Clinical Features										
		Histology	Smoking	PD-L1	ECOG	Bone Met.	Liver Met.	Brain Met.	T	N	M	Stage
Zero-shot	Accuracy (%)	0.87 [0.84, 0.89]	0.72 [0.69, 0.75]	0.76 [0.73, 0.79]	0.93 [0.91, 0.95]	0.84 [0.82, 0.87]	0.91 [0.88, 0.93]	0.91 [0.88, 0.93]	0.47 [0.43, 0.50]	0.50 [0.46, 0.54]	0.77 [0.74, 0.80]	0.78 [0.75, 0.81]
	F1 score	0,60 [0.56, 0.65]	0.71 [0.69, 0.77]	0.66 [0.64, 0.69]	0.87 [0.82, 0.92]	0.79 [0.75, 0.83]	0.74 [0.69, 0.80]	0.78 [0.73, 0.83]	0.27 [0.25, 0.37]	0.30 [0.27, 0.35]	0.64 [0.60, 0.68]	0.43 [0.40, 0.46]
	Precision (%)	0,73 [0.70, 0.77]	0.80 [0.75, 0.83]	0.85 [0.83, 0.87]	0.86 [0.80, 0.92]	0.85 [0.80, 0.89]	0.70 [0.62, 0.78]	0.74 [0.66, 0.80]	0.43 [0.24, 0.46]	0.40 [0.33, 0.47]	0.63 [0.60, 0.66]	0.42 [0.40, 0.44]
	Recall (%)	0,78 [0.75, 0.80]	0.68 [0.64, 0.72]	0.71 [0.70, 0.73]	0.88 [0.82, 0.93]	0.74 [0.68, 0.79]	0.79 [0.71, 0.86]	0.84 [0.77, 0.89]	0.29 [0.26, 0.32]	0.31 [0.28, 0.34]	0.82 [0.78, 0.86]	0.56 [0.53, 0.58]
Few-shot	Accuracy (%)	0.85 [0.82, 0.88]	0.82 [0.79, 0.84]	0.78 [0.75, 0.81]	0.94 [0.93, 0.96]	0.77 [0.74, 0.80]	0.91 [0.89 , 0.93]	0.91 [0.89, 0.93]	0.39 [0.36, 0.43]	0.51 [0.47, 0.54]	0.91 [0.89, 0.93]	0.90 [0.88, 0.92]
	F1 score	0.56 [0.51, 0.61]	0.79 [0.75, 0.82]	0.75 [0.72, 0.78]	0.90 [0.85, 0.94]	0.63 [0.57, 0.68]	0.71 [0.64, 0.78]	0.77 [0.71, 0.82]	0.22 [0.19, 0.25]	0.31 [0.27, 0.34]	0.77 [0.72, 0.82]	0.47 [0.43, 0.50]
	Precision (%)	0.72 [0.69, 0.75]	0.77 [0.73, 0.81]	0.84 [0.81, 0.87]	0.90 [0.84, 0.95]	0.91 [0.86, 0.96]	0.81 [0.73, 0.89]	0.82 [0.75, 0.88]	0.24 [0.20, 0.27]	0.40 [0.33, 0.46]	0.75 [0.69, 0.80]	0.47 [0.43, 0.52]
	Recall (%)	0.76 [0.74, 0.77]	0.82 [0.78, 0.86]	0.75 [0.73, 0.78]	0.90 [0.85, 0.94]	0.48 [0.42, 0.54]	0.63 [0.55, 0.72]	0.72 [0.65, 0.79]	0.24 [0.21, 0.27]	0.31 [0.28, 0.34]	0.81 [0.75, 0.86]	0.46 [0.42, 0.50]
Few-shot + clinical ann.	Accuracy (%)	0.91 [0.89, 0.93]	0.87 [0.84, 0.89]	0.83 [0.80, 0.85]	0.97 [0.95, 0.98]	0.82 [0.79, 0.85]	0.91 [0.89, 0.93]	0.90 [0.88, 0.93]	0.48 [0.45, 0.52]	0.43 [0.39, 0.47]	0.92 [0.90, 0.94]	0.91 [0.89, 0.93]
	F1 score	0.75 [0.69, 0.80]	0.85 [0.82, 0.88]	0.81 [0.78, 0.84]	0.95 [0.91, 0.97]	0.76 [0.71, 0.80]	0.73 [0.66, 0.79]	0.76 [0.70, 0.81]	0.20 [0.17, 0.23]	0.26 [0.22, 0.30]	0.73 [0.67, 0.79]	0.41 [0.36, 0.45]
	Precision (%)	0.72 [0.69, 0.76]	0.88 [0.85, 0.91]	0.86 [0.84, 0.89]	0.94 [0.89, 0.98]	0.85 [0.80, 0.89]	0.76 [0.68, 0.83]	0.77 [0.69, 0.83]	0.27 [0.22, 0.32]	0.42 [0.34, 0.49]	0.79 [0.71, 0.85]	0.52 [0.44, 0.59]
	Recall (%)	0.90 [0.80, 0.95]	0.83 [0.79, 0.87]	0.81 [0.78, 0.83]	0.95 [0.93, 0.97]	0.68 [0.63, 0.74]	0.71 [0.63, 0.79]	0.74 [0.67, 0.81]	0.22 [0.20, 0.24]	0.28 [0.25, 0.31]	0.70 [0.64, 0.75]	0.39 [0.36, 0.42]

Table S3.2: Mistral 7B performance for each feature extracted and prompting strategy (Italian) on the IO cohort.

Type of prompt	Metrics	Clinical Features										
		Histology	Smoking	PD-L1	ECOG	Bone Met.	Liver Met.	Brain Met.	T	N	M	Stage
Zero-shot	Accuracy (%)	0.85 [0.82, 0.88]	0.67 [0.64, 0.70]	0.81 [0.78, 0.84]	0.89 [0.86, 0.91]	0.79 [0.76,0.82]	0.80 [0.77, 0.83]	0.86 [0.83, 0.88]	0.24 [0.21, 0.27]	0.47 [0.44, 0.51]	0.68 [0.65, 0.71]	0.79 [0.76, 0.82]
	F1 score	0.54 [0.49, 0.59]	0.57 [0.53, 0.62]	0.78 [0.74, 0.80]	0.87 [0.84, 0.90]	0.67 [0.62, 0.72]	0.58 [0.52, 0.64]	0.70 [0.64, 0.75]	0.19 [0.16, 0.22]	0.35 [0.31, 0.39]	0.55 [0.51, 0.59]	0.42 [0.39, 0.45]
	Precision (%)	0.72 [0.69, 0.75]	0.64 [0.57, 0.71]	0.84 [0.81, 0.87]	0.85 [0.81, 0.89]	0.89 [0.84, 0.93]	0.46 [0.39, 0.52]	0.60 [0.53, 0.67]	0.33 [0.25, 0.40]	0.36 [0.33, 0.40]	0.57 [0.55, 0.60]	0.41 [0.39, 0.43]
	Recall (%)	0.75 [0.73, 0.76]	0.58 [0.55, 0.63]	0.79 [0.76, 0.81]	0.91 [0.88, 0.93]	0.54 [0.48, 0.60]	0.80 [0.72, 0.87]	0.84 [0.77, 0.90]	0.27 [0.21, 0.36]	0.41 [0.33, 0.54]	0.69 [0.63, 0.75]	0.49 [0.44, 0.53]
Few-shot	Accuracy (%)	0.77 [0.74, 0.80]	0.65 [0.62, 0.68]	0.89 [0.87, 0.92]	0.87 [0.84, 0.89]	0.77 [0.74, 0.81]	0.87 [0.84, 0.89]	0.85 [0.83, 0.88]	0.23 [0.20, 0.25]	0.46 [0.42, 0.50]	0.65 [0.62, 0.69]	0.85 [0.82, 0.87]
	F1 score	0.49 [0.47, 0.51]	0.56 [0.51, 0.61]	0.89 [0.87, 0.92]	0.86 (95% CI: [0.82, 0.89])	0.70 [0.66, 0.75]	0.67 [0.60, 0.73]	0.69 [0.63, 0.74]	0.16 [0.14, 0.18]	0.33 [0.30, 0.36]	0.54 [0.50, 0.57]	0.47 [0.42, 0.52]
	Precision (%)	0.72 [0.47, 0.74]	0.71 [0.62, 0.77]	0.90 [0.88, 0.92]	0.86 [0.84, 0.88]	0.74 [0.69, 0.80]	0.59 [0.52, 0.67]	0.60 [0.53, 0.67]	0.24 [0.21, 0.27]	0.34 [0.30, 0.36]	0.58 [0.55, 0.60]	0.46 [0.42, 0.50]
	Recall (%)	0.71 [0.69, 0.72]	0.59 [0.55, 0.62]	0.89 [0.87, 0.91]	0.89 [0.83, 0.92]	0.67 [0.61, 0.72]	0.77 [0.70, 0.85]	0.81 [0.74, 0.87]	0.23 [0.20, 0.26]	0.36 [0.32, 0.39]	0.71 [0.66, 0.77]	0.52 [0.42, 0.68]
Few-shot + clinical ann.	Accuracy (%)	0.88 [0.86, 0.90]	0.64 [0.60, 0.68]	0.83 [0.81, 0.86]	0.90 [0.88, 0.93]	0.45 [0.41, 0.49]	0.85 [0.82, 0.87]	0.87 [0.85, 0.90]	0.21 [0.18, 0.24]	0.48 [0.45, 0.52]	0.73 [0.69, 0.76]	0.84 [0.81, 0.86]
	F1 score	0.68 [0.63, 0.73]	0.59 [0.53, 0.63]	0.83 [0.81, 0.86]	0.88 [0.84, 0.91]	0.59 [0.55, 0.62]	0.63 [0.57, 0.70]	0.70 [0.64, 0.76]	0.17 [0.14, 0.19]	0.34 [0.30, 0.37]	0.58 [0.54, 0.62]	0.45 [0.40, 0.50]
	Precision (%)	0.68 [0.64, 0.71]	0.69 [0.62, 0.74]	0.84 [0.81, 0.86]	0.88 [0.86, 0.90]	0.42 [0.38, 0.46]	0.55 [0.47, 0.62]	0.67 [0.59, 0.74]	0.27 [0.24, 0.30]	0.35 [0.32, 0.39]	0.59 [0.56, 0.62]	0.51 [0.46, 0.57]
	Recall (%)	0.79 [0.65, 0.88]	0.59 [0.55, 0.63]	0.84 [0.81, 0.86]	0.89 [0.84, 0.93]	0.98 [0.96, 0.99]	0.76 [0.68, 0.83]	0.74 [0.66, 0.81]	0.22 [0.19, 0.25]	0.36 [0.32, 0.40]	0.72 [0.66, 0.78]	0.46 [0.37, 0.61]

Table S3.3: LlaMa 3.1 8B performance for each feature extracted and prompting strategy (Italian) on the TKI cohort.

Type of prompt	Metrics	Clinical Features										
		Histology	Smoking	PD-L1	ECOG	Bone Met.	Liver Met.	Brain Met.	T	N	M	Stage
Zero-shot	Accuracy (%)	0.98 [0.96, 1.00]	0.53 [0.46, 0.60]	0.64 [0.57, 0.70]	0.92 [0.88, 0.96]	0.89 [0.83, 0.92]	0.88 [0.82, 0.92]	0.83 [0.77, 0.88]	0,11 [0.06, 0.16]	0,21 [0.15, 0.27]	0,48 [0.40, 0.55]	0,27 [0.20, 0.33]
	F1 score	0.83 [0.53, 1.00]	0.51 [0.42, 0.59]	0.57 [0.52, 0.62]	0.9 [0.75, 0.96]	0.89 [0.84, 0.93]	0.6 [0.43, 0.74]	0.77 [0.69, 0.85]	0,1 [0.06, 0.14]	0,22 [0.12, 0.34]	0,26 [0.23, 0.30]	0,19 [0.15, 0.24]
	Precision (%)	0.94 [0.52, 1.00]	0.7 [0.64, 0.77]	0.54 [0.48, 0.59]	0.88 [0.77, 0.96]	0.86 [0.78, 0.92]	0.46 [0.31, 0.62]	0.66 [0.55, 0.76]	0,07 [0.04, 0.10]	0,14 [0.07, 0.26]	0,23 [0.19, 0.26]	0,46 [0.11, 0.49]
	Recall (%)	0.78 [0.53, 1.00]	0.51 [0.44, 0.58]	0.62 [0.57, 0.68]	0.93 [0.76, 0.96]	0.93 [0.85, 0.96]	0.85 [0.64, 0.95]	0.93 [0.83, 0.97]	0,25 [0.18, 0.32]	0,47 [0.29, 0.63]	0,44 [0.26, 0.61]	0,5 [0.27, 0.64]
Few-shot	Accuracy (%)	0.98 [0.96, 1.00]	0.84 [0.78, 0.89]	0.62 [0.55, 0.69]	0.94 [0.90, 0.97]	0.83 [0.77, 0.88]	0.9 [0.85, 0.94]	0.84 [0.78, 0.88]	0,1 [0.06, 0.14]	0,19 [0.14, 0.24]	0,54 [0.47, 0.61]	0,31 [0.24, 0.37]
	F1 score	0.95 [0.83, 1.00]	0.82 [0.76, 0.88]	0.58 [0.50, 0.65]	0.87 [0.76, 0.97]	0.82 [0.75, 0.88]	0.64 [0.45, 0.79]	0.76 [0.68, 0.83]	0,1 [0.05, 0.14]	0,28 [0.12, 0.38]	0,3 [0.23, 0.37]	0,24 [0.16, 0.32]
	Precision (%)	0.95 [0.81, 1.00]	0.86 [0.79, 0.91]	0.8 [0.69, 0.87]	0.87 [0.76, 0.97]	0.92 [0.84, 0.96]	0.53 [0.36, 0.70]	0.7 [0.58, 0.79]	0,07 [0.03, 0.11]	0,19 [0.07, 0.33]	0,23 [0.19, 0.29]	0,48 [0.11, 0.54]
	Recall (%)	0.95 [0.82, 1.00]	0.8 [0.74, 0.87]	0.57 [0.50, 0.64]	0.87 [0.77, 0.98]	0.73 [0.64, 0.81]	0.8 [0.58, 0.92]	0.84 [0.73, 0.91]	0,24 [0.15, 0.33]	0,57 [0.29, 0.64]	0,48 [0.30, 0.65]	0,55 [0.32, 0.67]
Few-shot + clinical ann.	Accuracy (%)	0.99 [0.97, 1.00]	0.78 [0.72, 0.83	0.68 [0.61, 0.75]	0.95 [0.92, 0.98]	0.89 [0.83, 0.92]	0.86 [0.80, 0.90]	0.85 [0.80, 0.90]	0,1 [0.06, 0.15]	0,2 [0.14, 0.26]	0,57 [0.50, 0.64]	0,32 [0.25, 0.39]
	F1 score	0.62 [0.54, 1.00]	0.78 [0.71, 0.84]	0.66 [0.58, 0.73]	0.92 [0.78, 0.98]	0.89 [0.84, 0.93]	0.55 [0.38, 0.69]	0.79 [0.71, 0.87]	0,06 [0.03, 0.09]	0,24 [0.10, 0.36]	0,25 [0.23, 0.26]	0,17 [0.14, 0.26]

	Precision (%)	0.62 [0.52, 1.00]	0.84 [0.79, 0.89]	0.81 [0.73, 0.88]	0.91 [0.79, 0.98]	0.88 [0.80, 0.93]	0.42 [0.28, 0.58]	0.7 [0.59, 0.79]	0,25 [0.02, 0.29]	0,42 [0.08, 0.58]	0,2 [0.17, 0.22]	0,44 [0.10, 0.65]
	Recall (%)	0.62 [0.54, 1.00]	0.76 [0.69, 0.82]	0.65 [0.58, 0.71]	0.95 [0.77, 0.98]	0.89 [0.83, 0.93]	0.8 [0.58, 0.92]	0.91 [0.81, 0.96]	0,21 [0.20, 0.24]	0,43 [0.27, 0.59]	0,33 [0.32, 0.33]	0,34 [0.33, 0.51]

Table S3.4: Mistral 7B performance for each feature extracted and prompting strategy (Italian) on the TKI cohort.

Type of prompt	Metrics	Clinical Features										
		Histology	Smoking	PD-L1	ECOG	Bone Met.	Liver Met.	Brain Met.	T	N	M	Stage
Zero-shot	Accuracy (%)	0.97 [0.94, 0.99]	0.56 [0.48, 0.63]	0.68 [0.61, 0.75]	0.9 [0.85, 0.94]	0.81 [0.75, 0.86]	0.89 [0.84, 0.93]	0.84 [0.79, 0.89]	0,24 [0.18, 0.30]	0,2 [0.13, 0.27]	0,28 [0.22, 0.35]	0,29 [0.23, 0.35]
	F1 score	0.5 [0.33, 0.87]	0.46 [0.38, 0.52]	0.62 [0.56, 0.69]	0.88 [0.74, 0.94]	0.79 [0.48, 0.50]	0.64 [0.48, 0.78]	0.79 [0.71, 0.86]	0,14 [0.09, 0.17]	0,2 [0.13, 0.27]	0,2 [0.16, 0.23]	0,25 [0.19, 0.30]
	Precision (%)	0.44 [0.33, 0.80]	0.55 [0.38, 0.71]	0.79 [0.74, 0.85]	0.88 [0.76, 0.95]	0.9 [0.46, 0.50]	0.5 [0.34, 0.66]	0.68 [0.46, 0.50]	0,2 [0.17, 0.24]	0,31 [0.22, 0.39]	0,23 [0.19, 0.26]	0,41 [0.33, 0.47]
	Recall (%)	0.66 [0.32, 0.99]	0.51 [0.44, 0.58]	0.66 [0.59, 0.72]	0.9 [0.73, 0.94]	0.7 [0.49, 0.50]	0.9 [0.70, 0.97]	0.93 [0.49, 0.50]	0,22 [0.16, 0.28]	0,51 [0.21, 0.61]	0,41 [0.15, 0.51]	0,59 [0.26, 0.64]
Few-shot	Accuracy (%)	0.99 [0.98, 1.00]	0.55 [0.48, 0.62]	0.74 [0.68, 0.80]	0.74 [0.67, 0.80]	0.83 [0.77, 0.88]	0.91 [0.86, 0.95]	0.87 [0.82, 0.91]	0,16 [0.11, 0.21]	0,22 [0.15, 0.28]	0,36 [0.29, 0.44]	0,42 [0.35, 0.49]
	F1 score	0.56 [0.33, 1.00]	0.45 [0.39, 0.52]	0.71 [0.64, 0.77]	0.64 [0.58, 0.79]	0.82 [0.76, 0.88]	0.68 [0.50, 0.81]	0.81 [0.72, 0.87	0,1 [0.06, 0.14]	0,22 [0.14, 0.30]	0,24 [0.20, 0.27]	0,37 [0.27, 0.46]
	Precision (%)	0.5 [0.33, 1.00]	0.56 [0.39, 0.72]	0.82 [0.76, 0.88]	0.73 [0.70, 0.89]	0.86 [0.77, 0.92]	0.57 [0.68, 0.87]	0.75 [0.63, 0.84]	0,21 [0.18, 0.24]	0,33 [0.24, 0.40]	0,24 [0.21, 0.27]	0.47 [0.40, 0.54]
	Recall (%)	0.67 [0.33, 1.00]	0.52 [0.45, 0.59]	0.69 [0.61, 0.76]	0.61 [0.55, 0.76]	0.79 [0.69, 0.86]	0.85 [0.79, 0.96]	0.88 [0.77, 0.94]	0,22 [0.14, 0.34]	0,54 [0.25, 0.62]	0,53 [0.49, 0.56]	0,71 [0.37, 0.73]
Few-shot + clinical ann.	Accuracy (%)	0,98 [0.96, 1.00]	0,47 [0.40, 0.54]	0,74 [0.67, 0.81]	0,76 [0.70, 0.82]	0.67 [0.60, 0.74]	0.88 [0.83, 0.92])	0.88 [0.82, 0.92]	0,32 [0.25, 0.39]	0,24 [0.18, 0.30]	0,44 [0.37, 0.52]	0,46 [0.39, 0.53]
	F1 score	0,5 [0.33, 1.00]	0,42 [0.35, 0.48]	0,71 [0.62, 0.79]	0,66 [0.60, 0.79]	0.75 [0.57, 0.70]	0.58 [0.41, 0.72]	0.8 [0.72, 0.87]	0,18 [0.13, 0.24]	0,23 [0.16, 0.30]	0,25 [0.21, 0.29]	0,44 [0.29, 0.57]

	Precision (%)	0,44 [0.33, 1.00]	0,55 [0.39, 0.66]	0,79 [0.71, 0.87]	0,75 [0.72, 0.90]	0.61 [0.69, 0.81]	0.47 [0.31, 0.64]	0.78 [0.66, 0.87]	0,24 [0.18, 0.33]	0,39 [0.32, 0.45]	0,22 [0.19, 0.25]	0,5 [0.38, 0.63]
	Recall (%)	0,66 [0.33, 1.00]	0,5 [0.43, 0.56]	0,67 [0.59, 0.75]	0,62 [0.57, 0.76]	0.96 [0.62, 0.72	0.75 [0.53, 0.89]	0.82 [0.71, 0.90]	0,28 [0.17, 0.40]	0,54 [0.22, 0.61]	0,42 [0.24, 0.59]	0,61 [0.37, 0.75]

S4 Language experiment

Table S4.1: Performances of LlaMa 3.1 8B for each feature extracted and language of zero-shot prompting. **P*value of the McNemar test performed between the contingency tables for prompts in Italian (Ita) and in English (Eng).

Metrics	Clinical Features																					
	Histology		Smoking		PD-L1		ECOG		Bone Met.		Liver Met.		Brain Met.		T		N		M		Stage	
	Eng	Ita	Eng	Ita	Eng	Ita	Eng	Ita	Eng	Ita	Eng	Ita	Eng	Ita	Eng	Ita	Eng	Ita	Eng	Ita	Eng	Ita
Accuracy (%)	0,86 [0.84, 0.89]	0.87 [0.84, 0.89]	0.48 [0.45, 0.52]	0.72 [0.69, 0.75]	0,92 [0.90, 0.94]	0.76 [0.73, 0.79]	0,95 [0.93, 0.97]	0.93 [0.91, 0.95]	0.83 [0.80, 0.85]	0.84 [0.82, 0.87]	0,88 [0.86, 0.91]	0.91 [0.88, 0.93]	0.90 [0.8, 0.93]	0.91 [0.88, 0.93]	0.48 [0.45, 0.52]	0.47 [0.43, 0.50]	0.55 [0.51, 0.59]	0.50 [0.46, 0.54]	0.69 [0.66, 0.72]	0.77 [0.74, 0.80]	0.70 [0.67, 0.73]	0.78 [0.75, 0.81]
F1 score	0,61 [0.51, 0.70]	0,60 [0.56, 0.65]	0.36 [0.33, 0.40]	0.71 [0.69, 0.77]	0,92 [0.90, 0.94]	0.66 [0.64, 0.69]	0,93 [0.90, 0.96]	0.87 [0.82, 0.92]	0.75 [0.71, 0.79]	0.79 [0.75, 0.83]	0,71 [0.65, 0.76]	0.74 [0.69, 0.80]	0.77 [0.73, 0.82]	0.78 [0.73, 0.83]	0.31 [0.28, 0.35]	0.27 [0.25, 0.37]	0.36 [0.33, 0.42]	0.30 [0.27, 0.35]	0.58 [0.54, 0.62]	0.64 [0.60, 0.68]	0.38 [0.36, 0.41]	0.43 [0.40, 0.46]
Precision (%)	0,78 [0.67, 0.93]	0,73 [0.70, 0.77]	0.71 [0.46, 0.72]	0.80 [0.75, 0.83]	0,92 [0.90, 0.94]	0.85 [0.83, 0.87]	0,94 [0.88, 0.97]	0.86 [0.80, 0.92]	0.88 [0.84, 0.93]	0.85 [0.80, 0.89]	0,63 [0.56, 0.71]	0.70 [0.62, 0.78]	0.73 [0.66, 0.80]	0.74 [0.66, 0.80]	0.43 [0.30, 0.50]	0.43 [0.24, 0.46]	0.40 [0.36, 0.46]	0.40 [0.33, 0.47]	0.61 [0.58, 0.63]	0.63 [0.60, 0.66]	0.40 [0.38, 0.42]	0.42 [0.40, 0.44]
Recall (%)	0,6 [0.51, 0.73]	0,78 [0.75, 0.80]	0.52 [0.50, 0.55]	0.68 [0.64, 0.72]	0,91 [0.89, 0.93]	0.71 [0.70, 0.73]	0,94 [0.91, 0.96]	0.88 [0.82, 0.93]	0.65 [0.60, 0.71]	0.74 [0.68, 0.79]	0,80 [0.73, 0.87]	0.79 [0.71, 0.86]	0.83 [0.77, 0.89]	0.84 [0.77, 0.89]	0.32 [0.29, 0.35]	0.29 [0.26, 0.32]	0.36 [0.32, 0.41]	0.31 [0.28, 0.34]	0.80 [0.75, 0.83]	0.82 [0.78, 0.86]	0.52 [0.49, 0.55]	0.56 [0.53, 0.58]
p value	<0.01		<0.01		<0.01		0.98		0.11		<0.05		0,61		<0.01		<0.01		<0.01		<0.01	

Table S4.2: Performances of LlaMa 3.1 8B for each feature extracted and language of few-shot prompting. **P*value of the McNemar test performed between the contingency tables for prompts in Italian (Ita) and in English (Eng).

Metrics	Clinical Features																					
	Histology		Smoking		PD-L1		ECOG		Bone Met.		Liver Met.		Brain Met.		T		N		M		Stage	
	Eng	Ita	Eng	Ita	Eng	Ita	Eng	Ita	Eng	Ita	Eng	Ita	Eng	Ita	Eng	Ita	Eng	Ita	Eng	Ita	Eng	Ita
Accuracy (%)	0.86 [0.83, 0.88]	0.85[0.82, 0.88]	0.59 [0.56, 0.63]	0.82 [0.79, 0.84]	0.75 [0.71, 0.78]	0.78 [0.75, 0.81]	0.95 [0.93, 0.96]	0.94 [0.93, 0.96]	0,67 [0.63, 0.70]	0.77 [0.74, 0.80]	0,89 [0.87, 0.91]	0.91 [0.89 , 0.93]	0,90 [0.88, 0.92]	0.91 [0.89, 0.93]	0.35 [0.32, 0.39]	0.39 [0.36, 0.43]	0.53 [0.49, 0.56]	0.51 [0.47, 0.54]	0.88 [0.85, 0.90]	0.91 [0.89, 0.93]	0.87 [0.85, 0.90]	0.90 [0.88, 0.92]
F1 score	0.52 [0.47, 0.57]	0.56 [0.51, 0.61]	0.54 [0.50, 0.57]	0.79 [0.75, 0.82]	0.66 [0.64, 0.68]	0.75 [0.72, 0.78]	0.92 [0.88, 0.95]	0.90 [0.85, 0.94]	0,31 [0.25, 0.37]	0.63 [0.57, 0.68]	0,60 [0.53, 0.68]	0.71 [0.64, 0.78]	0,75 [0.69, 0.80]	0.77 [0.71, 0.82]	0.22 [0.19, 0.25]	0.22 [0.19, 0.25]	0.32 [0.29, 0.37]	0.31 [0.27, 0.34]	0.73 [0.68, 0.77]	0.77 [0.72, 0.82]	0.49 [0.46, 0.53]	0.47 [0.43, 0.50]
Precision (%)	0.47 [0.44, 0.51]	0.72 [0.69, 0.75]	0.69 [0.67, 0.72]	0.77 [0.73, 0.81]	0.88 [0.86, 0.89]	0.84 [0.81, 0.87]	0.91 [0.85, 0.95]	0.90 [0.84, 0.95]	0,93 [0.86, 1.00]	0.91 [0.86, 0.96]	0,79 [0.70, 0.88]	0.81 [0.73, 0.89]	0,75 [0.68, 0.83]	0.82 [0.75, 0.88]	0.42 [0.32, 0.53]	0.24 [0.20, 0.27]	0.40 [0.35, 0.46]	0.40 [0.33, 0.46]	0.69 [0.65, 0.74]	0.75 [0.69, 0.80]	0.47 [0.44, 0.50]	0.47 [0.43, 0.52]
Recall (%)	0.66 [0.54, 0.74]	0.76 [0.74, 0.77]	0.64 [0.61, 0.67]	0.82 [0.78, 0.86]	0.70 [0.69, 0.72]	0.75 [0.73, 0.78]	0.94 [0.92, 0.96]	0.90 [0.85, 0.94]	0,19 [0.14, 0.23]	0.48 [0.42, 0.54]	0,49 [0.40, 0.58]	0.63 [0.55, 0.72]	0,74 [0.66, 0.81]	0.72 [0.65, 0.79]	0.25 [0.22, 0.27]	0.24 [0.21, 0.27]	0.33 [0.30, 0.38]	0.31 [0.28, 0.34]	0.80 [0.74, 0.85]	0.81 [0.75, 0.86]	0.54 [0.50, 0.57]	0.46 [0.42, 0.50]
p value	<0.05		<0.01		<0.01		0.33		0.11		<0.01		0.08		<0.01		<0.01		<0.01		<0.01	

S5 Agreement analysis

Table S5.1: Fleiss *k* agreement with 95% CI for each feature extracted (Italian).

Feature	Llama zero vs Llama few	Llama zero vs Llama few + ann.	Llama zero vs Mistral zero	Llama zero vs Mistral few	Llama zero vs Mistral few + ann.	Llama few vs Mistral zero	Llama few vs Mistral few	Llama few vs Mistral few + ann.	Llama few vs Llama few + ann.	Llama few + ann. vs Mistral zero	Llama few + ann. vs Mistral few	Llama few + ann. vs Mistral	Mistral zero vs Mistral few	Mistral zero vs Mistral few + ann.	Mistral few vs Mistral few + ann.

												few + ann.			
Histology	0.80 [0.75, 0.84]	0.64 [0.59, 0.69]	0.78 [0.74, 0.83]	0.67 [0.62, 0.72]	0.67 [0.62, 0.72]	0.75 [0.70, 0.80]	0.65 [0.60, 0.70]	0.67 [0.63, 0.72]	0.63 [0.58, 0.68]	0.59 [0.54, 0.63]	0.55 [0.51, 0.60]	0.74 [0.69, 0.78]	0.72 [0.67, 0.76]	0.69 [0.64, 0.75]	0.64 [0.60, 0.69]
Smoking	0.53 [0.47, 0.58]	0.64 [0.59, 0.69]	0.29 [0.23, 0.34]	0.26 [0.21, 0.31]	0.31 [0.26, 0.37]	0.55 [0.50, 0.60]	0.54 [0.49, 0.60]	0.50 [0.45, 0.54]	0.75 [0.70, 0.79]	0.44 [0.39, 0.49]	0.44 [0.39, 0.49]	0.42 [0.36, 0.47]	0.67 [0.62, 0.72]	0.51 [0.46, 0.56]	0.59 [0.54, 0.64]
PD-L1	0.77 [0.73, 0.81]	0.77 [0.72, 0.80]	0.72 [0.68, 0.76]	0.64 [0.59, 0.68]	0.53 [0.49, 0.58]	0.68 [0.63, 0.72]	0.69 [0.64, 0.73]	0.59 [0.55, 0.63]	0.85 [0.82, 0.88]	0.71 [0.67, 0.75]	0.74 [0.70, 0.78]	0.66 [0.61, 0.70]	0.75 [0.71, 0.79]	0.65 [0.61, 0.69]	0.82 [0.78, 0.85]
ECOG	0.92 [0.89, 0.94]	0.90 [0.88, 0.93]	0.77 [0.73, 0.81]	0.76 [0.71, 0.79]	0.81 [0.77, 0.84]	0.81 [0.77, 0.82]	0.78 [0.75, 0.82]	0.82 [0.769 0.86]	0.94 [0.92, 0,96]	0.80 [0.77, 0.84]	0.78 [0.74, 0.81]	0.83 [0.79, 0.86]	0.85 [0.82, 0.88]	0.86 [0.82, 0.89]	0.87 [0.84, 0.90]
Bone Met	0.66 [0.60, 0.72]	0.86 [0.82, 0.90]	0.65 [0.59, 0.71]	0.64 [0.58, 0.70]	-0.27 [-0.33, -0.20]	0.56 [0.48, 0.63]	0.53 [0.46, 0.60]	-0.47 [-0.53, -0.41]	0.71 [0.65, 0.77]	0.65 [0.59, 0.71]	0.65 [0.59, 0.71]	-0.30 [-0.36, -0.24]	0.64 [0.58, 0.70]	-0.41 [-0.48, -0.35]	-0.24 [-0.31, -0.17]
Liver Met	0.77 [0.71, 0.84]	0.84 [0.78, 0.89]	0.60 [0.52, 0.66]	0.73 [0.66, 0.79]	0.73 [0.66, 0.79]	0.47 [0.39, 0.54]	0.63 [0.55, 0.70]	0.58 [0.50, 0.65]	0.88 [0.83, 0.93]	0.53 [0.45, 0.60]	0.66 [0.59, 0.73]	0.64 [0.56, 0.70]	0.72 [0.66, 0.77]	0.72 [0.66, 0.77]	0.78 [0.73, 0.83]
Brain Met	0.85 [0.80, 0.89]	0.89 [0.84, 0.93]	0.77 [0.71, 0.82]	0.72 [0.65, 0.77]	0.79 [0.73, 0.84]	0.69 [0.62, 0.75]	0.67 [0.61, 0.74]	0.75 [0.68, 0.81]	0.92 [0.87, 0.95]	0.73 [0.67, 0.79]	0.68 [0.60, 0.73]	0.76 [0.70, 0.82]	0.80 [0.75, 0.84]	0.79 [0.74, 0.84]	0.78 [0.72, 0.83]
T	0.46 [0.41, 0.51]	0.15 [0.09, 0.21]	0.04 [-0.01, 0.08]	0.01 [-0.03, 0.06]	0.04 [0.00, 0.08]	0.11 [0.06, 0.16]	0.09 [0.05, 0.14]	0.09 [0.05, 0.14]	0.03 [-0.04, 0.09]	-0.27 [-0.32, -0.22]	-0.28 [-0.32, -0.23]	-0.24 [-0.28, -0.19]	0.48 [0.43, 0.53]	0.40 [0.34, 0.45]	0.49 [0.44, 0.55]
N	0.65 [0.59, 0.70]	0.45 [0.38, 0.51]	0.16 [0.11, 0.22]	0.22 [0.17, 0.27]	0.15 [0.10, 0.21]	0.21 [0.15, 0.26]	0.24 [0.18, 0.29]	0.21 [0.15, 0.26]	0.35 [0.28, 0.41]	-0.01 [-0.06, 0.04]	0.07 [0.02, 0.12]	-0.04 [-0.09, 0.02]	0.52 [0.47, 0.56]	0.45 [0.40, 0.50]	0.48 [0.43, 0.52]
M	0.45 [0.37, 0.53]	0.20 [0.12, 0.29]	0.36 [0.29, 0.43]	0.34 [0.27, 0.42]	0.35 [0.28, 0.43]	0.24 [0.15, 0.31]	0.23 [0.15, 0.31]	0.27 [0.19, 0.34]	0.65 [0.54, 0.74]	0.08 [0.00, 0.16]	0.05 [-0.03, 0.13]	0.13 [0.05, 0.21]	0.54 [0.48, 0.60]	0.53 [0.46, 0.60]	0.57 [0.51, 0.63]
Stage	0.30 [0.22, 0.39]	0.03 [-0.04, 0.10]	0.38 [0.31, 0.45]	0.23 [0.15, 0.30]	0.09 [0.03, 0.16]	0.29 [0.21, 0.38]	0.34 [0.23, 0.43]	0.26 [0.16, 0.35]	0.48 [0.35, 0.60]	0.07 [-0.01, 0.15]	0.24 [0.13, 0.34]	0.20 [0.10, 0.31]	0.39 [0.31, 0.47]	0.26 [0.18, 0.33]	0.46 [0.37, 0.55]
Median (Q1, Q3)	0.66 [0.50, 0.79]	0.64 [0.33, 0.85]	0.60 [0.32, 0.75]	0.64 [0.25, 0.70]	0.35 [0.12, 0.7]	0.55 [0.27, 0.69]	0.54 [0.29, 0.66]	0.50 [0.24, 0.63]	0.71 [0.55, 0.87]	0.53 [0.08, 0.68]	0.55 [0.16, 0.67]	0.42 [0.05, 0.70]	0.67 [0.53, 0.74]	0.53 [0.43, 0.71]	0.59 [0.49, 0.78]

Table S5.2: Results of Wilcoxon signed-rank test used to compare agreement between two sets of methods and FDR to correct for multiple comparisons.

Reference	Method A	Method B	Raw p-value	FDR p-value
Mistral zero	Llama few	Llama few + ann.	0.10	0.16
Mistral zero	Llama few	Mistral few	0.001	0.009
Mistral zero	Llama few	Llama zero	0.36	0.47
Mistral zero	Llama few	Mistral few + ann.	0.36	0.47
Mistral zero	Llama few + ann.	Mistral few	0.001	0.009
Mistral zero	Llama few + ann.	Llama zero	0.03	0.06
Mistral zero	Llama few + ann.	Mistral few + ann.	0.08	0.14
Mistral zero	Mistral few	Llama zero	0.01	0.04
Mistral zero	Mistral few	Mistral few + ann.	0.002	0.01
Mistral zero	Llama zero	Mistral few + ann.	0.41	0.51
Llama few	Mistral zero	Llama few + ann.	0.004	0.01
Llama few	Mistral zero	Mistral few	0.76	0.80
Llama few	Mistral zero	Llama zero	0.002	0.01
Llama few	Mistral zero	Mistral few + ann.	0.46	0.55
Llama few	Llama few + ann.	Mistral few	0.004	0.01
Llama few	Llama few + ann.	Llama zero	0.63	0.72
Llama few	Llama few + ann.	Mistral few + ann.	0.004	0.014
Llama few	Mistral few	Llama zero	0.004	0.014
Llama few	Mistral few	Mistral few + ann.	0.24	0.36
Llama few	Llama zero	Mistral few + ann.	0.0009	0.009
Llama few + ann.	Mistral zero	Llama few	0.0009	0.009
Llama few + ann.	Mistral zero	Mistral few	0.83	0.85
Llama few + ann.	Mistral zero	Llama zero	0.001	0.009
Llama few + ann.	Mistral zero	Mistral few + ann.	0.32	0.44
Llama few + ann.	Llama few	Mistral few	0.0009	0.009
Llama few + ann.	Llama few	Llama zero	0.46	0.55
Llama few + ann.	Llama few	Mistral few + ann.	0.001	0.009

Llama few + ann.	Mistral few	Llama zero	0.01	0.04
Llama few + ann.	Mistral few	Mistral few + ann.	1.0	1.0
Llama few + ann.	Llama zero	Mistral few + ann.	0.03	0.06
Mistral few	Mistral zero	Llama few	0.0009	0.009
Mistral few	Mistral zero	Llama few + ann.	0.002	0.01
Mistral few	Mistral zero	Llama zero	0.002	0.01
Mistral few	Mistral zero	Mistral few + ann.	0.76	0.80
Mistral few	Llama few	Llama few + ann.	0.14	0.23
Mistral few	Llama few	Llama zero	0.89	0.91
Mistral few	Llama few	Mistral few + ann.	0.06	0.12
Mistral few	Llama few + ann.	Llama zero	0.32	0.44
Mistral few	Llama few + ann.	Mistral few + ann.	0.05	0.10
Mistral few	Llama zero	Mistral few + ann.	0.06	0.12
Llama zero	Mistral zero	Llama few	0.006	0.01
Llama zero	Mistral zero	Llama few + ann.	0.36	0.47
Llama zero	Mistral zero	Mistral few	0.17	0.26
Llama zero	Mistral zero	Mistral few + ann.	0.41	0.51
Llama zero	Llama few	Llama few + ann.	0.27	0.40
Llama zero	Llama few	Mistral few	0.0009	0.009
Llama zero	Llama few	Mistral few + ann.	0.0009	0.009
Llama zero	Llama few + ann.	Mistral few	0.10	0.16
Llama zero	Llama few + ann.	Mistral few + ann.	0.04	0.08
Llama zero	Mistral few	Mistral few + ann.	0.70	0.77
Mistral few + ann.	Mistral zero	Llama few	0.001	0.009
Mistral few + ann.	Mistral zero	Llama few + ann.	0.08	0.14
Mistral few + ann.	Mistral zero	Mistral few	0.01	0.03
Mistral few + ann.	Mistral zero	Llama zero	0.02	0.05
Mistral few + ann.	Llama few	Llama few + ann.	0.57	0.66
Mistral few + ann.	Llama few	Mistral few	0.001	0.009
Mistral few + ann.	Llama few	Llama zero	0.76	0.80
Mistral few + ann.	Llama few + ann.	Mistral few	0.006	0.01
Mistral few + ann.	Llama few + ann.	Llama zero	0.57	0.66

Mistral few + ann.	Mistral few	Llama zero	0.006	0.01

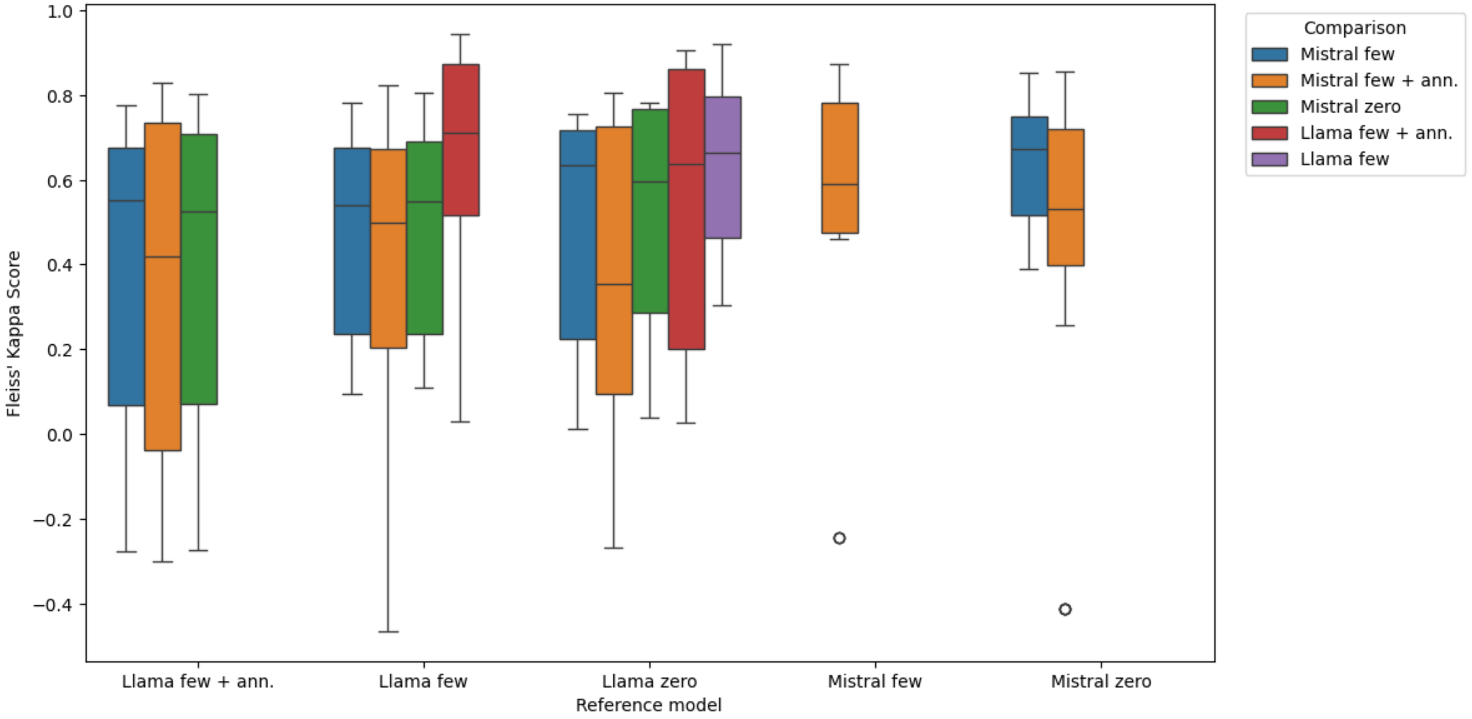

Figure S5.3 Boxplot showing the pairwise agreement of LLMs (LLaMa 3.1 8B and Mistral) with different Italian prompting strategies. Each box represents the distribution of Fleiss' Kappa values calculated across all cancer-related features for each model-prompt pair.

S6 Missing information analysis

Missing information analysis

As additional analysis, we investigated the hallucination rate associated with the inference of MI (i.e., the “non mentionated” label), under both zero-shot and few-shot+ann prompting. For instance, under zero-shot conditions (Table S6.1) we observed that, for smoking and ECOG PS with LLaMa 3.1, the hallucination rate is 48.09% and 32.84%, respectively (vs 33.5% and 9.42%in the GT). With Mistral and smoking and ECOG PS, the hallucination rate decreases to 9.79% and 13.43%, proving that this model tends to hallucinate less towards the “not mentionated” label. However, under a few-shot+ann design (Table S6.2), the rate of hallucination reduces to18.05.% and 19.4% with LLaMa 3.1, respectively. This analysis demonstrates that the inference of real missing information is improved with few-shot + ann prompting. Finally, in supplementary Figures S6.3 and S6.4 we report examples of MI inference for 5 patients’ cases in which PD-L1<1% has been wrongly inferred as “not mentioned”. These examples align with the main analysis proving better extraction with few-shot+ann.

Table S6.1: Analysis of missing value information in zero-shot prompting.

		**Llama 3.1 8B**		**Mistral 7B**	
**Clinical Features**	MI (GT). (n/%)	Number of matches	Halluc. (n/%)	Number of matches	Halluc. (n/%)
**Histology**	6 (0.84%)	6	0 (0.00%)	6	0 (0.00%)
**Smoking**	235 (33.5%)	122	113 (48.09%)	212	23 (9.79%)
**PD-L1**	205 (28.83%)	193	7 (3.41%)	184	14 (6.83%)
**ECOG PS**	67 (9.42%)	45	22 (32.84%)	58	9 (13.43%)
**T (TNM)**	5 (0.70%)	0	5 (100%)	1	4 (100%)
**N (TNM)**	4 (0.56%)	0	4 (100%)	1	3 (100%)
**Stage**	5 (0.70%)	0	5 (100%)	0	5 (100%)

Table S6.2: Analysis of missing value information in few-shot +ann data.

		Llama 3.1 8B		Mistral 7B	
**Clinical Features**	MI (GT). (n/%)	Number of matches	Halluc. (n/%)	Number of matches	Halluc. (n/%)
**Histology**	6 (0.84%)	5	1 (16.67%)	4	2 (33.33%)
**Smoking**	235 (33.5%)	198	37 (18.05%)	138	97 (41.28%)
**PD-L1**	205 (28.83%)	193	7 (3.41%)	148	52 (25.37%)
**ECOG PS**	67 (9.42%)	54	13 (19.40%)	52	14 (20.90%)
**T (TNM)**	5 (0.74%)	0	5 (100%)	1	5 (100%)
**N (TNM)**	4 (0.56%)	0	4 (100%)	0	4 (100%)
**Stage**	5 (0.74%)	0	5 (100%)	1	4(100%)

S7 Medical expertise experiment

Table S7.1 Fleiss *k* agreement with 95% CI for each feature extracted in the zero-shot phase of the experiment.

Feature	Experienced	Resident	Student	Experienced vs student	Resident vs Student	Experienced vs Resident
Bone Met	0.79 [0.79, 0.80]	0.71 [0.71, 0.72]	0.54 [0.54, 0.55]	0.67 [0.67, 0.68]	0.63 [0.63, 0.64]	0.75 [0.74, 0.75]
Brain Met	0.88 [0.87, 0.88]	0,75 [0.74, 0.75]	0.88 [0.88, 0.89]	0.88 [0.87, 0.88]	0.81 [0.81, 0.82]	0.82 [0.81, 0.82]
Liver Met	0.82 [0.81, 0.82]	0.70 [0.69, 0.70]	0.70 [0.69, 0.70]	0.76 [0.75, 0.76]	0.69 [0.68, 0.69]	0.76 [0.75, 0.76]
T	0.18 [0.18, 0.19]	0.25 [0.25, 0.26]	0.32 [0.31, 0.32]	0.24 [0.24, 0.25]	0.30 [0.29, 0.30]	0.23 [0.22, 0.23]

N	0.35 [0.35, 0.36]	0.34 [0.33, 0.35]	0.40 [0.39, 0.41]	0.37 [0.37, 0.38]	0.38 [0.37, 0.38]	0.36 [0.35, 0.36]
M	0.44 [0.44, 0.45]	0.21 [0.21, 0.22]	0.37 [0.37, 0.38]	0.41 [0.40, 0.41]	0.32 [0.31, 0.32]	0.34 [0.34, 0.35]
Stage	0.28 [0.28, 0.29]	0.22 [0.22, 0.23]	0.46 [0.45, 0.46]	0.36 [0.35, 0.36]	0.33 [0.33, 0.34]	0.33 [0.33, 0.34]
Median (Q1, Q3)	0.44 [0.32, 0.81]	0.34 [0.24, 0.7]	0.46 [0.38, 0.62]	0.41[0.36, 0.72]	0.38 [0.32, 0.66]	0.36 [0.34, 0.76]

Table S7.2 Fleiss *k* agreement with 95% CI for each feature extracted in the few-shot phase of the experiment.

Feature	Experienced	Resident	Student	Experienced vs student	Resident vs Student	Experienced vs Resident
Bone Met	0.79 [0.79, 0.80]	0.53 [0.53, 0.54]	0.64 [0.63, 0.64]	0.71 [0.70, 0.71]	0.57 [0.56, 0.57]	0.68 [0.67, 0.68]
Brain Met	0.79 [0.79, 0.80]	0.47 [0.46, 0.47]	0.77 [0.77, 0.77]	0.78 [0.77, 0.78]	0.61 [0.60, 0.61]	0.64 [0.64, 0.64]
Liver Met	0.90 [0.90, 0.90]	0.67 [0.66, 0.67]	0.86 [0.85, 0.86]	0.87 [0.87, 0.88]	0.76 [0.76, 0.76]	0.80 [0.80, 0.80]
T	0.35 [0.35, 0.36]	0.37 [0.36, 0.38]	0.51 [0.50, 0.51]	0.43 [0.42, 0.43]	0.45 [0.44, 0.45]	0.37 [0.37, 0.37]
N	0.24 [0.23, 0.24]	0.35 [0.34, 0.36]	0.45 [0.44, 0.46]	0.33 [0.33, 0.34]	0.41 [0.41, 0.42]	0.30 [0.29, 0.30]
M	0.17 [0.16, 0.17]	0.22 [0.21, 0.22]	0.50 [0.49, 0.50]	0.29 [0.28, 0.29]	0.35 [0.34, 0.35]	0.22 [0.21, 0.22]
Stage	0.08 [0.08, 0.09]	0.21 [0.20, 0.22]	0.38 [0.37, 0.38]	0.21 [0.20, 0.21]	0.30 [0.29, 0.30]	0.16 [0.16, 0.16]
Median (Q1, Q3)	0.35 [0.20, 0.79]	0.37 [0.29, 0.50]	0.51 [0.48, 0.70]	0.43 [0.31, 0.74]	0.45 [0.38, 0.59]	0.37 [0.26, 0.66]

Table S7.3 Results of Wilcoxon rank test used to compare the intra and inter-rater Fleiss's *k* agreement between the two phases of the experiments.

Group	Wilcoxon p-value
experienced	0.08
resident	0.29

student	0.03
experienced vs student	0.46
resident vs student	0.81
experienced vs resident	0.04

S8 TKI cohort results

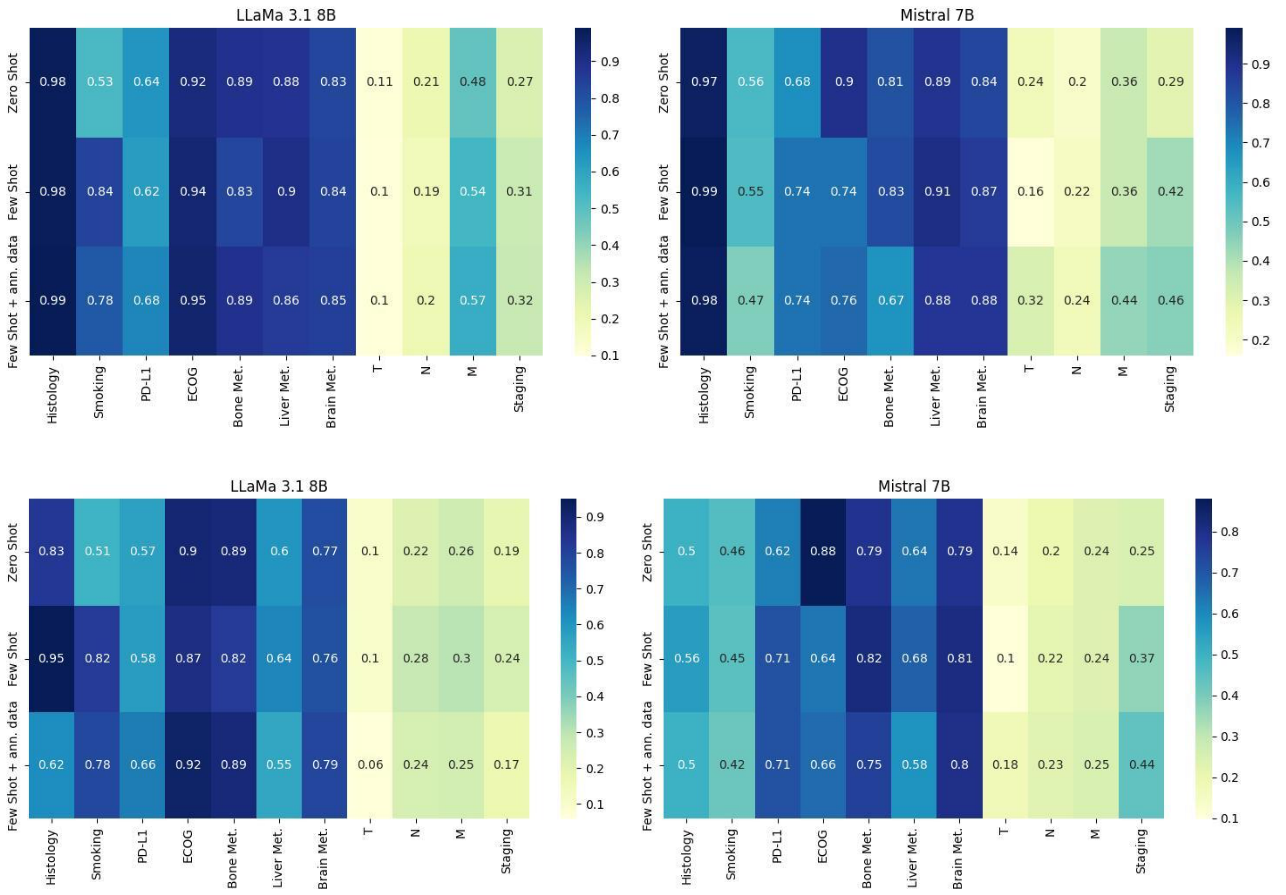

Figure S8.1: Heatmaps of a) accuracy and b) F1-Score for LLaMa 3.1 8B and Mistral 7B by prompting technique on the TKI cohort.
